# Double jersey finger: A systematic review and case series

**DOI:** 10.1016/j.jpra.2026.03.005

**Published:** 2026-03-16

**Authors:** Bryan Lim, Ishith Seth, Yi Xie, Gianluca Marcaccini, Emilio Trignano, Roberto Cuomo, Warren M. Rozen

**Affiliations:** a Department of Plastic Surgery, Peninsula Health, Melbourne, Victoria, Australia; b Department of Plastic, Hand & Faciomaxillary Surgery, Alfred Health, Melbourne, Victoria, Australia; c Plastic and Reconstructive Surgery, Department of Medicine, Surgery and Neuroscience, University of Siena, Siena, Italy; d Department of Medicine, Surgery and Pharmacy, University of Sassari, Italy

**Keywords:** Jersey finger, Closed avulsion injury, Flexor tendon rupture, Flexor digitorum profundus, Leddy and Packer

## Abstract

**Introduction:**

Closed flexor tendon avulsions typically affect the flexor digitorum profundus (FDP) from hyperextension of a flexed finger. Avulsion of both the FDP and flexor digitorum superficialis (FDS) in the same finger is rare and complicates surgery and prognosis.

**Methods:**

This case study details a simultaneous FDP and FDS rupture, emphasizing accurate diagnosis and surgery. A systematic review of English case studies on presentation, diagnosis, treatment, and outcomes was conducted, following 2020 PRISMA guidelines.

**Case series:**

A 40-year-old male developed jersey finger following an assault, and a 26-year-old male sustained a similar injury while playing Australian Rules football. Both had simultaneous FDP and FDS tendon avulsions successfully treated with micro-Mitek suture anchors and early active mobilization.

**Results:**

The PRISMA flow diagram started with 70 records, 7 duplicates were removed, and 26 were excluded for measuring unintended outcomes, leaving 37 for screening. After excluding 14 cases that focused on single tendon ruptures, 23 were assessed for eligibility. Seven more were removed due to translation issues or lack of access, resulting in 16 studies included in the review.

**Discussion:**

This case documents a successful primary repair over 3 weeks post-injury. A review of 24 cases highlights the advantages of using micro-Mitek suture anchors for repair, which enhance strength and early mobilization for better functional recovery.

**Conclusion:**

This manuscript underscores the importance of personalized surgical planning, timing, techniques and comprehensive postoperative rehabilitation in managing rare and complex hand injuries. Further research is needed to refine management strategies.

## Introduction

Closed flexor tendon avulsions typically involving the flexor digitorum profundus (FDP) tendon, colloquially known as “jersey finger”.^1^^,^^2^ This usually results from forced hyperextension against a flexed finger, often seen in contact sports such as rugby or American football. However, the simultaneous closed avulsion of both the FDP and the flexor digitorum superficialis (FDS) tendons in the same digit is exceedingly rare with limited cases documented in the literature.^1^^,^^3^, ^4^, ^5^, ^6^, ^7^, ^8^, ^9^, ^10^, ^11^, ^12^, ^13^, ^14^, ^15^, ^16^, ^17^, ^18^ The complexity of these injuries is further compounded by the zone of rupture, which significantly influences the surgical approach and the prognosis.

Traditionally, the management of closed flexor tendon avulsions emphasized prompt surgical intervention within the first 2 weeks post-injury to optimize outcomes.^9^^,^^19^ Beyond this period, the formation of adhesions and retraction of the tendon stumps often necessitate a two-stage reconstruction approach.^8^^,^^14^ Despite this, there are instances where delayed primary repair has been attempted with varying degrees of success.^9^ However, the rarity of dual tendon avulsions and the optimal timing for surgical repair have been subjects of ongoing debate.

We discuss the use of Mitek anchor sutures for the primary repair of FDS and FDP tendons, the challenges encountered during the delayed intervention, and the postoperative rehabilitation protocol that facilitated a favorable outcome. Additionally, we review existing literature on similar cases, aiming to enhance the current discourse on combined FDP and FDS ruptures. This report highlights the potential for successful outcomes in delayed primary repairs of dual tendon avulsions, advocating for individualized surgical planning and the importance of expert intraoperative decision-making.

## Methods

This case series involved two patients who suffered combined FDP and FDS tendons ruptures. This will underscore the significance of meticulous diagnostic and surgical techniques in hand surgery. Subsequently, these cases will lead to a systematic literature review that explores diverse methodologies employed for diagnosing and treating such conditions.

### Systematic review

To find the relevant case studies to compare to, a systematic approach focused on simultaneous closed avulsion of both FDP and FDS tendons was conducted, detailing the clinical presentation, diagnostic challenges, and treatment outcomes. A detailed search strategy was employed, utilizing PubMed, Web of Science, EMBASE, and the Cochrane Library databases. Keywords and phrases used in the search included “flexor tendon avulsion”, “jersey finger”, “simultaneous avulsion”, “hand surgery”, “case study” and “case series”. The search included studies published from the inception of these databases through April 2025, following the 2020 PRISMA reporting guidelines (Supplementary Figure 1).

### Selection criteria

The search was restricted to articles published in English, and additional sources were identified through the reference lists of the articles found. The inclusion criteria for the study were broad to ensure a comprehensive collection of relevant data. They encompassed all studies, including case reports, case series, observational studies, and reviews focusing on simultaneously closed avulsion of FDP and FDS tendons. Patients aged 16 and older of any gender diagnosed with this type of injury were included, and the reports had to detail clinical presentation (including mechanism of injury, site rupture and involved digit), diagnostic methods (including imaging and surgical confirmation), treatment approaches, and outcomes where available. There were no geographical restrictions for the studies. Conversely, the exclusion criteria comprised articles not focusing on simultaneously closed avulsion of FDP and FDS tendon, and studies not available in English or without a sufficient English abstract. Additionally, studies reporting the same cases or data already included in other publications were omitted to avoid duplication. Studies reporting patients under 16 years of age were also excluded.

Data Extraction from the selected articles was extracted by two independent reviewers and cross-checked for accuracy. The data included author, year of publication, patient demographics, clinical presentation, diagnostic methods, treatment provided, and postoperative outcomes. The findings were then collated into a suitable table (Table 1). This discussion delved into the potential reasons for the rarity of simultaneous closed avulsions of both FDP and FDS tendons, highlighted the diagnostic challenges, and provided recommendations for future clinical practice.

**Table 1:** Literature on closed injuries of both FDS and FDP tendons.

Author	Age (y)	Sex	Mechanism	Involved digit	Site rupture	Technique	Time to surgery	Follow-up	Outcome
Lim et al. (this case series)	40	M	Assault	Ring finger	FDP: zone 1 FDS: zone 2 at insertion time	Micro-Mitek anchor primary repair	4 weeks	2 months	Full ROM at MCP, PIP, DIP
	26	M	Jersey finger	Ring finger	FDP: zone 3 FDS: zone 2	Transosseous technique with 3–0 Prolene in a four-strand Adelaide configuration, secured over a button on the nail plate; FDS repaired with two 3–0 mini Mitek anchor sutures; reinforced with 4–0 PDS epitendinous sutures	Later that night	N/A	N/A
de Villenueve Bargemon et al.	30	M	Jersey finger	Middle finger	FDP: zone 1 FDS: zone 1	A Brunner approach was used, necessitating a cut back; the FDS was resected, and the FDP anchored with a steel wire.	0 days	6 months	Full ROM at MCP, PIP, DIP
Boyes et al.	Multiple (5 cases, ages not given)	Multiple (5 cases, sex not given)	Hyperextension × 4 Jersey finger × 1	Multiple (5 cases, finger not given)	FDP: zone 1 FDS: zone 2 Both at insertions in all 5 cases	Not described	N/A	N/A	N/A
Cheung and Chow	24	M	Jersey finger	Ring finger	FDP: zone 1 FDS: zone 2 Both at insertions	Both tendons sutured to periosteal flap. FDP reinforced w/pull-out suture	4 days	3.5 months	Full ROM MCP and PIP, DIP
Cañadas Moreno et al.	16	M	Blast	Index and middle fingers	FDP: zone 1 FDS: zone 2 Both at insertions	FDP: pull-out suture FDS: anchor suture	0 days	4 months	DIP flexion: 30 degrees × 2 digits
Toussaint et al.	23	M	Blast	Ring and little fingers	FDP: dilacerated in zone 1 + volar plate pull-out, FDS: zone 2 Both at insertions	FDP: pull-out suture FDS: resected	1 day	7 months	D4: DIP flexion 15; PIP flexion: 10 D5: DIP 40 PIP flexion: 10
Backe and Posner	23	M	Hyperextension	Ring finger	FDP: zone 1 FDS: zone 2 Both at insertions	Palmaris longus tendon graft	4 weeks	NM	Complete extension and active flexion within 1.5 cm of midpalmar crease DIP stiffness
Lanzetta and Conolly	28	M	Hyperextension	Ring finger	FDP: zone 1 FDS: zone 2 Both at insertions	Two stage repair: excision both tendons, left palmaris tendon graft 9 weeks post first surgery	3 days	4 months	Full ROM at MCP, PIP, DIP
Johnson and Colville	35	M	Hyperextension	Ring finger	FDP: zone 2 FDS: zone 2 Both at insertions	FDP: intraosseous repair FDS: resected	4 weeks	5 months	E/F–MCP: 0/82 PIP: 4/102 DIP: 10/20 Tip to distal palmar crease = 0 cm
Jordan et al.	20	M	Jersey finger	Middle finger	FDP: zone 1 FDS: zone 2 Both at insertions	FDP: pull-out suture FDS: resected	14 days	4 months	Full movement of PIP and arc of 20–70 at DIPJ
Soro et al.	30	M	Jersey finger	Little finger	FDP: zone 1 FDS: zone 3 FDP at insertion FDS midsubstance	FDP: pull-out suture FDS: tendon graft	0 days	6 months	E/F–MCP: 0/95 PIP: 10/85 DIP: 10/22 Tip to distal palmar crease = 0 cm
Oğün et al.	21	M	Jersey finger	Ring finger	FDP: zone 1 FDS: zone 2 Both midsubstance	FDP: pull-out suture FDS: resected	0 days	19 months	TAM = 230 degrees PIP and DIP stiffness
Naohito et al.	49	M	Direct shock	Little finger	FDP: zone 2 FDS: zone 2 Both midsubstance	FDP: end-to-end suture, FDS: resected	20 days	4 months	E/F–MCP: 30/0/80 PIP 0/40/85 DIP: 0/5/60
Matthews and Walton.	28	M	Repeated microtrauma	Middle finger	FDP: zone 2, FDS: zone 2 Both midsubstance	2 stage repair: silicone rod, reoperation at 10 weeks, PL graft	14 days	3.5 months	Full ROM at MCP, PIP, DIP
Cuggy et al.	27	M	Jersey finger	Ring finger	FDP: zone 3 FDS: zone 3	2 micro-Mitek suture anchors and modified Kessler suture	2 days	3.25 months	Achieved flexion to the midpalmar crease MCPJ:0 to 100 degrees PIPJ: −8 to 98 degrees DIPJ: −6 to 40 degrees Grip strength: 58 % of the contralateral side
Estrella and Gavino	20s	F	Laceration	Index finger	FDP: zone 2 FDS: zone 2	WALANT, Brunner incision, four-core cruciate tendon repair was performed using 4–0 non-absorbable suture with 6–0 non-absorbable circumferential suture on the FDP, and a modified Kessler suture technique on the FDS.	N/A	3 weeks	Regained 60–70 degrees flexion at the MCPJ
Thitiworakarn et al. (2022)	65	M	Strong grip while grasping a sickle	Index finger	Zone 3	FDS used as tendon graft for FDP (modified Kessler core 4-strand)	1 month	N/A	Nearly full motion, returned to normal activity
	45	F	Strong grip while grasping a shovel	Little finger	Zone 3	FDP repaired with modified Kessler + epitendinous sutures	2 months	N/A	Nearly full motion, returned to normal activity
	56	M	Strong grip while digging	Little finger	Zone 3	FDP and FDS repaired with modified Kessler + epitendinous sutures	5 weeks	N/A	Nearly full motion, returned to normal activity
	79	M	Strong grip while digging with a hoe	Little finger	Zone 3	FDP repaired with 4-strand core suture	1 week	N/A	Nearly full motion, returned to normal activity

Abbreviations: DIP, distal interphalangeal; DIPJ, DIP joint; E/F, extension/flexion; FDP, flexor digitorum profundus; FDS, flexor digitorum superficialis; M, male; MCP, metacarpophalangeal joint; N/A, not available; NM, not mentioned; PIP, proximal interphalangeal; ROM, range of motion; TAM, total active motion.

### Case report 1

**Figure 1.1:**
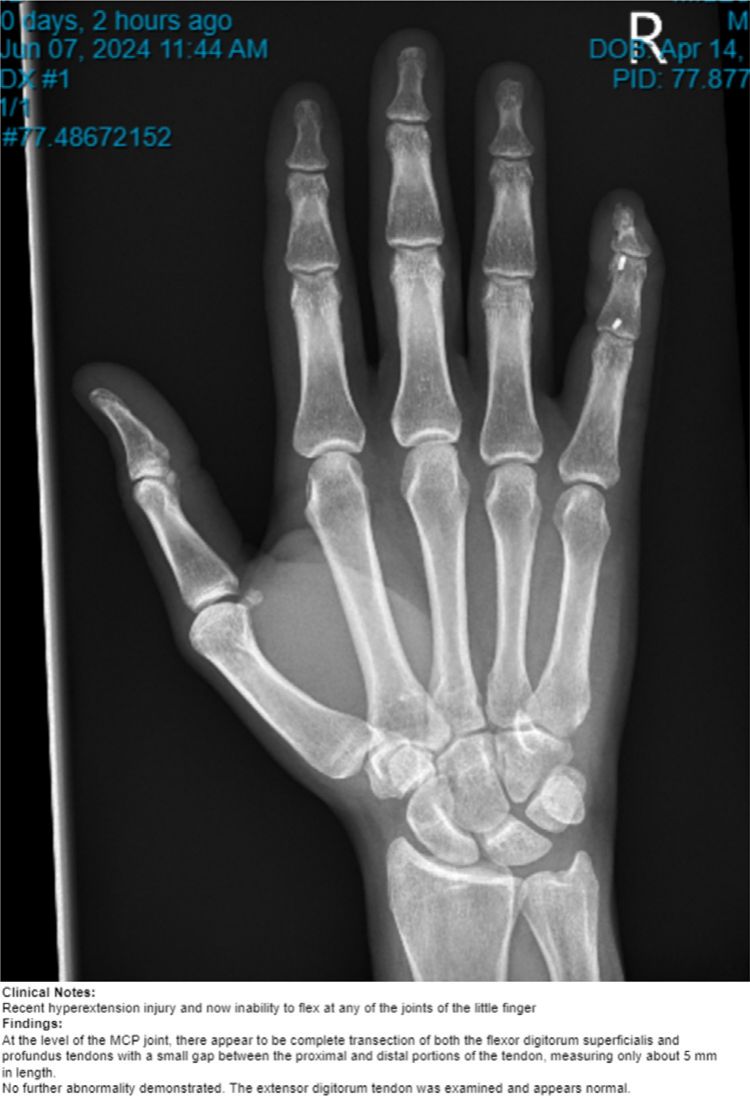
Frontal X-Ray of right hand (Case 1).

**Figure 1.2:**
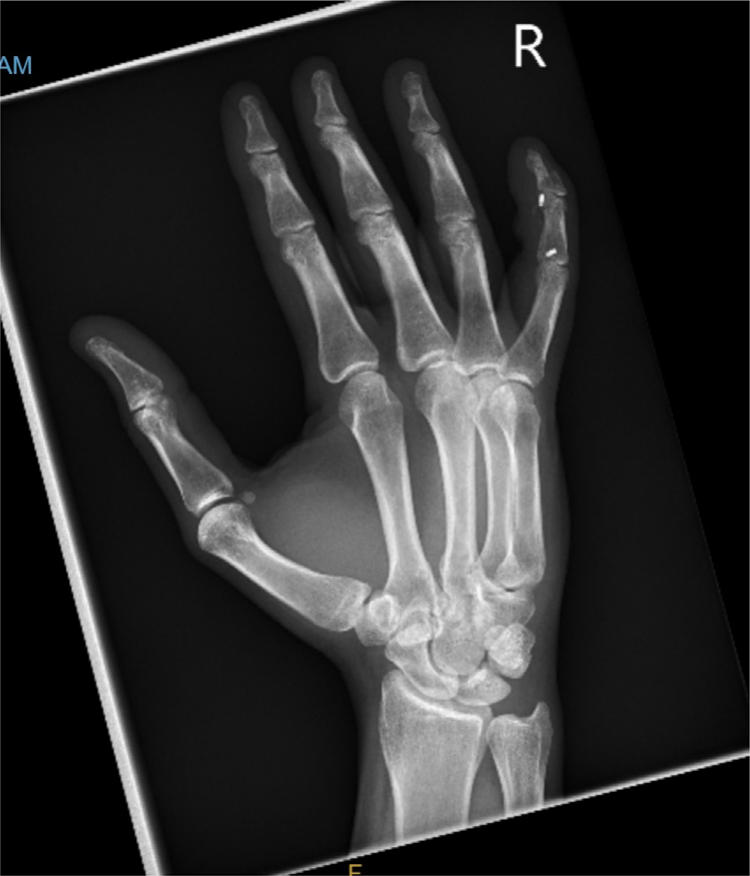
Oblique X-Ray of right hand (Case 1).

**Figure 1.3:**
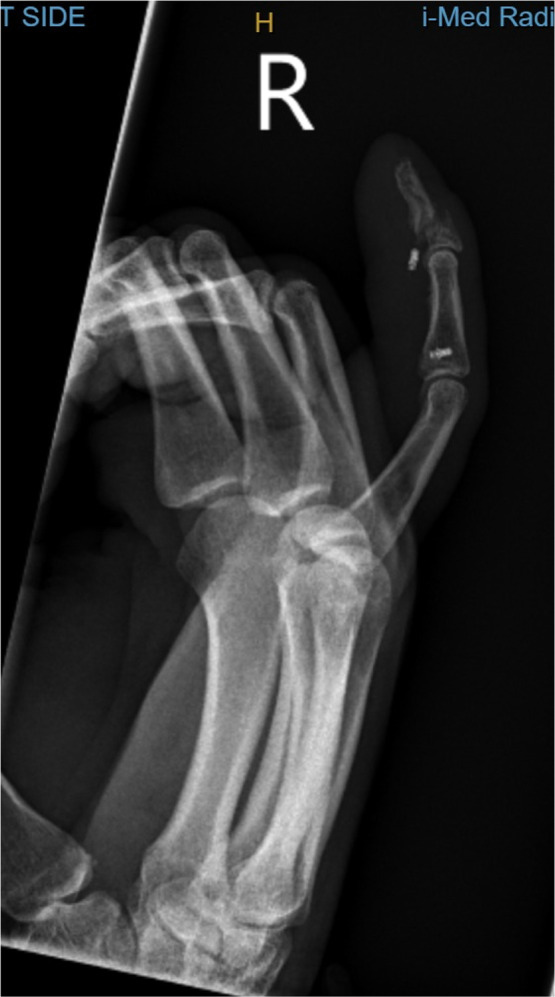
Lateral X-Ray of right hand (Case 1).

**Figure 2.1:**
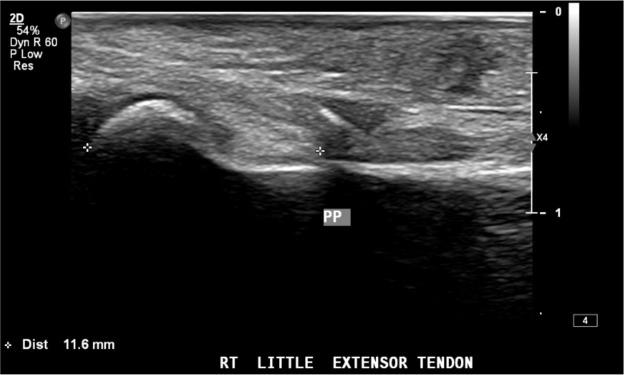
Ultrasound of flexor tendons (longitudinal, Case 1).

**Figure 2.2:**
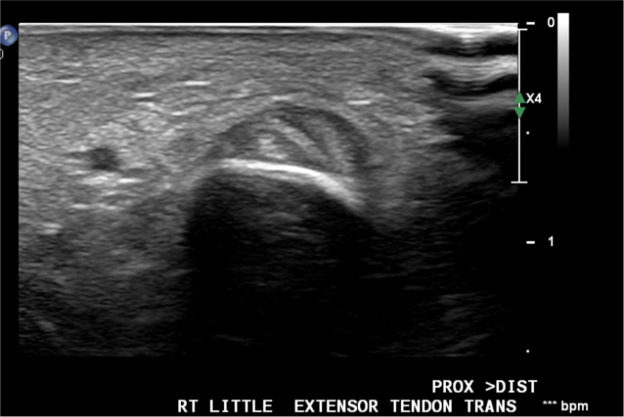
Ultrasound of flexor tendons (transverse, Case 1).

A 40-year-old right-hand dominant male with no significant past medical history nor regular medications presented to the emergency department 4 weeks after an assault, reporting an inability to flex the ring finger of his left hand. The injury occurred during the altercation, where he caught his left ring finger in another person’s shirt, immediately experiencing pain followed by swelling. Upon examination, the patient’s ring finger was swollen and tender, held in extension at rest, and exhibiting an abnormal cascade. Isolating both the FDS and FDP, active flexion was absent in both the distal and proximal interphalangeal joints, although normal passive range of motion was preserved. A palpable mass was detected just proximal to the level of the A1 pulley, indicating the presence of one or both flexor tendons in zone III. X-ray radiographs revealed no bony injury (Figure 1.1, Figure 1.2–1.3). The ultrasound images are illustrated in Figure 2.1, Figure 2.2. Clinical judgment suggested an FDP avulsion, potentially accompanied by an FDS rupture. Due to the rarity of this presentation, an ultrasound scan was conducted, confirming the avulsion of both FDS and FDP tendons retracted to the metacarpophalangeal joint (MCPJ).

Surgical exploration with Bruner’s incisions revealed avulsion of both the FDP and FDS tendons at their insertion points, alongside a rupture of the A4 pulley, while both volar plates remained intact. The tendons were retrieved from the palm using curved mosquito forceps. The two FDS tendon slips were reattached to their middle phalanx insertion sites using two micro-Mitek suture anchors (DePuy Synthes) with the Adelaide technique. The FDP tendon was repositioned through the chiasm and inserted into the base of the distal phalanx with two micro-Mitek suture anchors and the Adelaide technique to each slip. The A4 pulley was not repaired as the remaining pulley system was intact, preventing bowstringing. Postoperatively, the patient was dressed and placed in a dorsal blocking splint in the intrinsic plus position following closure with nonabsorbable sutures. Both tendons demonstrated good glide intraoperatively, with no gapping. The patient commenced an early active mobilization protocol per the Manchester Rehabilitation Protocol for flexor tendon lesions on day 3 with hand therapists. At this stage, he transitioned to a thermoplastic dorsal Duran splint. Active flexion commenced at 3–4 weeks, with light strengthening introduced at 6 weeks. The splint was discontinued at 6 weeks, and unrestricted strengthening was allowed from 8 to 10 weeks. Follow-up assessments were performed at 1 week, 2 weeks, 6 weeks, 3 months, and 6 months postoperatively. By 6 months, the patient demonstrated excellent functional recovery, achieving the following active range of motion in the affected finger: MCPJ 0–85°, PIPJ 0–105°, and DIPJ 0–70°, with minimal (0–5°) terminal extension lag at the PIPJ and DIPJ. The total active motion (TAM) was 260°, representing approximately 95 % of the contralateral hand. The patient reported full return to daily activities and work tasks without pain, stiffness, or functional limitation.

### Case report 2

A 26-year-old male Australian football player presented to the emergency department with left ring finger pain following an injury sustained during a game. The patient reported that his left ring finger became caught in an opposing player’s jumper. He experienced an immediate dislocation at the distal interphalangeal joint (DIPJ), which he self-relocated. Despite reduction, he had persistent pain over the DIPJ and presented to the hospital later that night.

**Figure 3:**
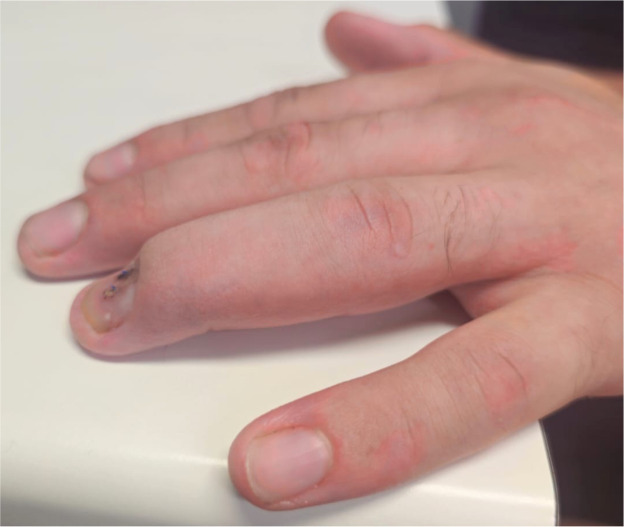
Left ring finger before surgical correction (Case 2).

**Figure 4.1:**
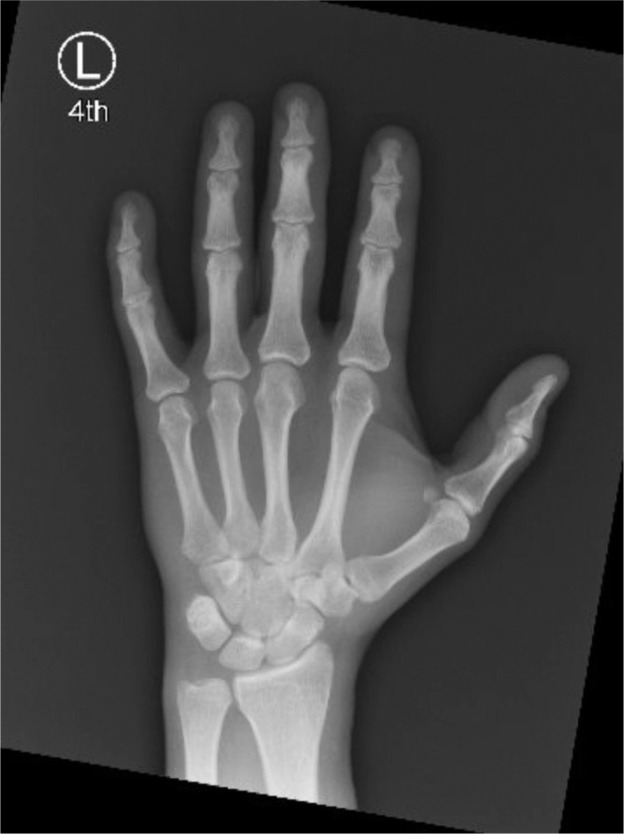
Frontal X-Ray of Left hand (Case 2: Pre-op).

**Figure 4.2:**
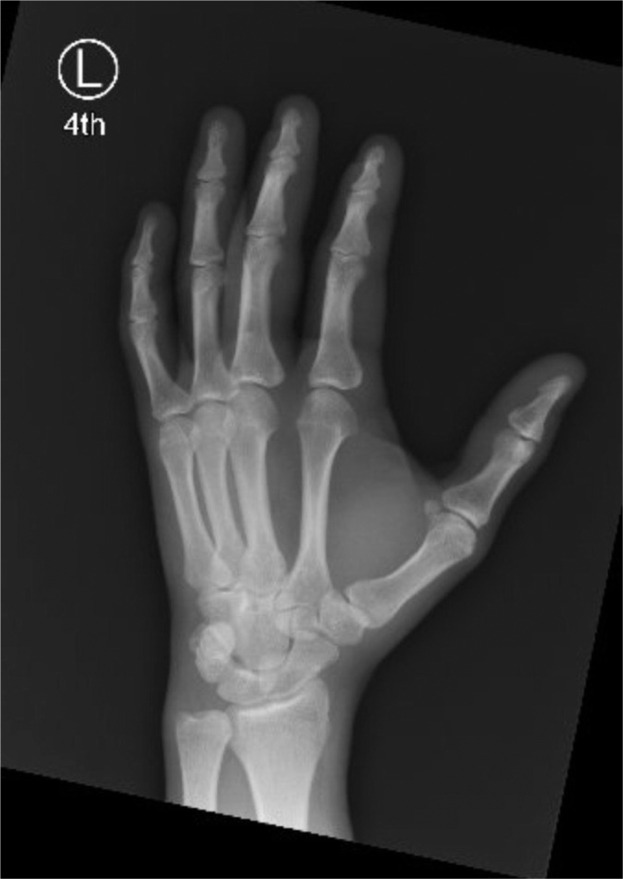
Oblique X-Ray of right hand (Case 2: Pre-op).

**Figure 4.3:**
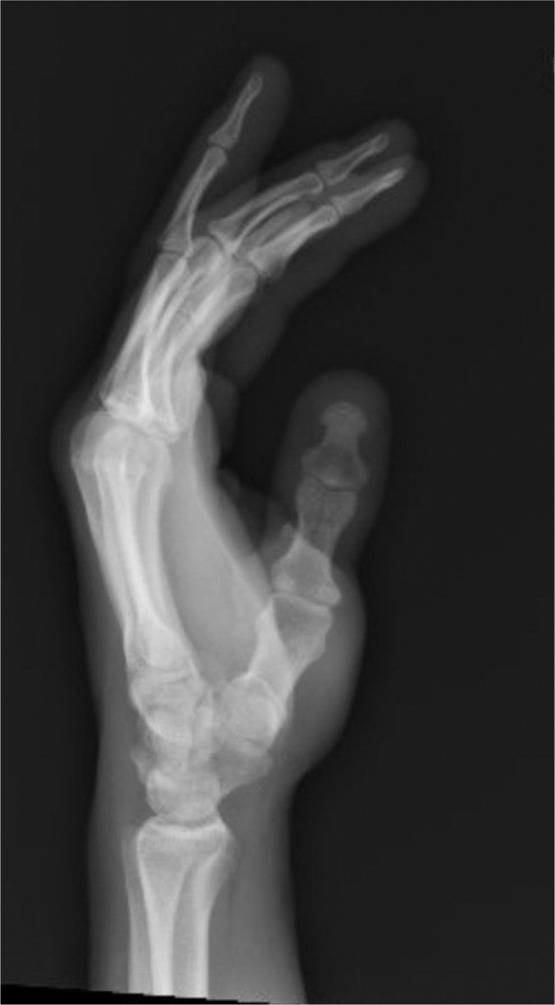
Lateral X-Ray of right hand (Case 2: Pre-op).

Upon examination, the injury was found to be closed (Figure 3). Tenderness extending from the MCPJ to the DIPJ suggested that one or both tendons had retracted into zone III, similar to the findings in the first case. The patient exhibited a limited range of motion in both flexion and extension. X-Ray revealed no bony injuries or fractures (Figure 4.1, Figure 4.2–4.3).

**Figure 5.1:**
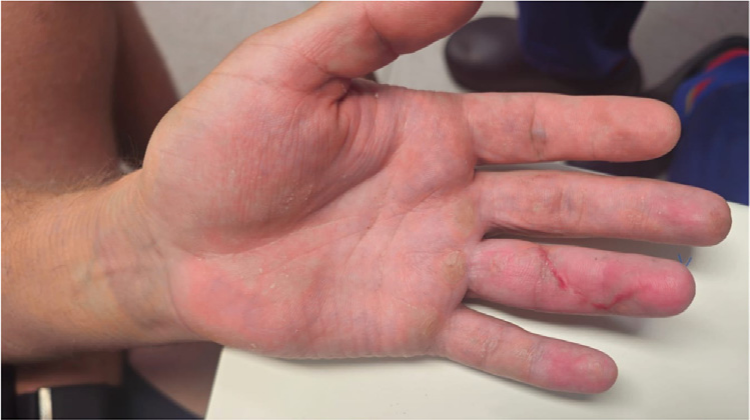
Left ring finger post-surgical correction (Case 2).

**Figure 5.2:**
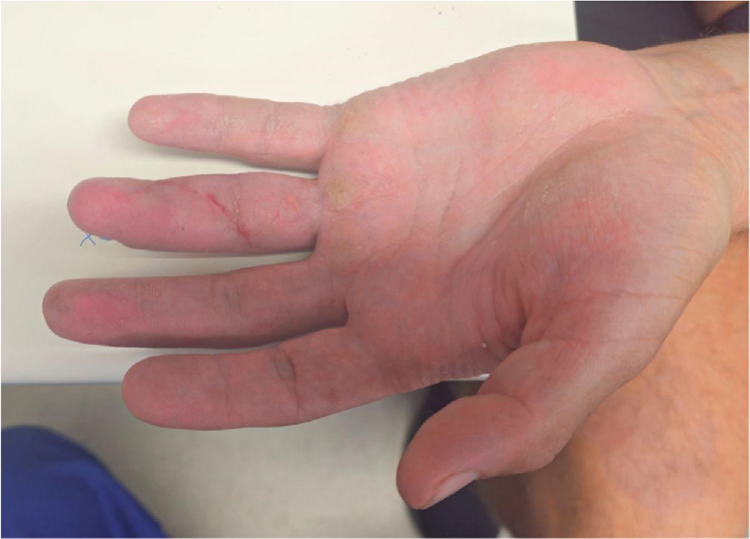
Left ring finger post-surgical correction - Lateral view (Case 2).

**Figure 5.3:**
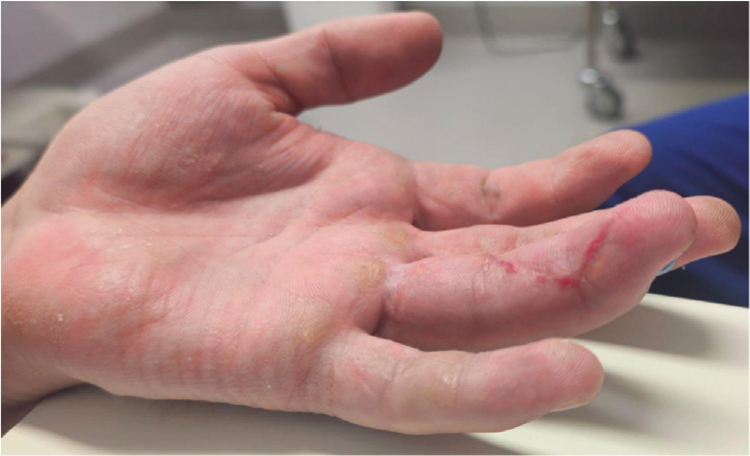
Left ring finger post-surgical correction - Medial view (Case 2).

**Figure 6.1:**
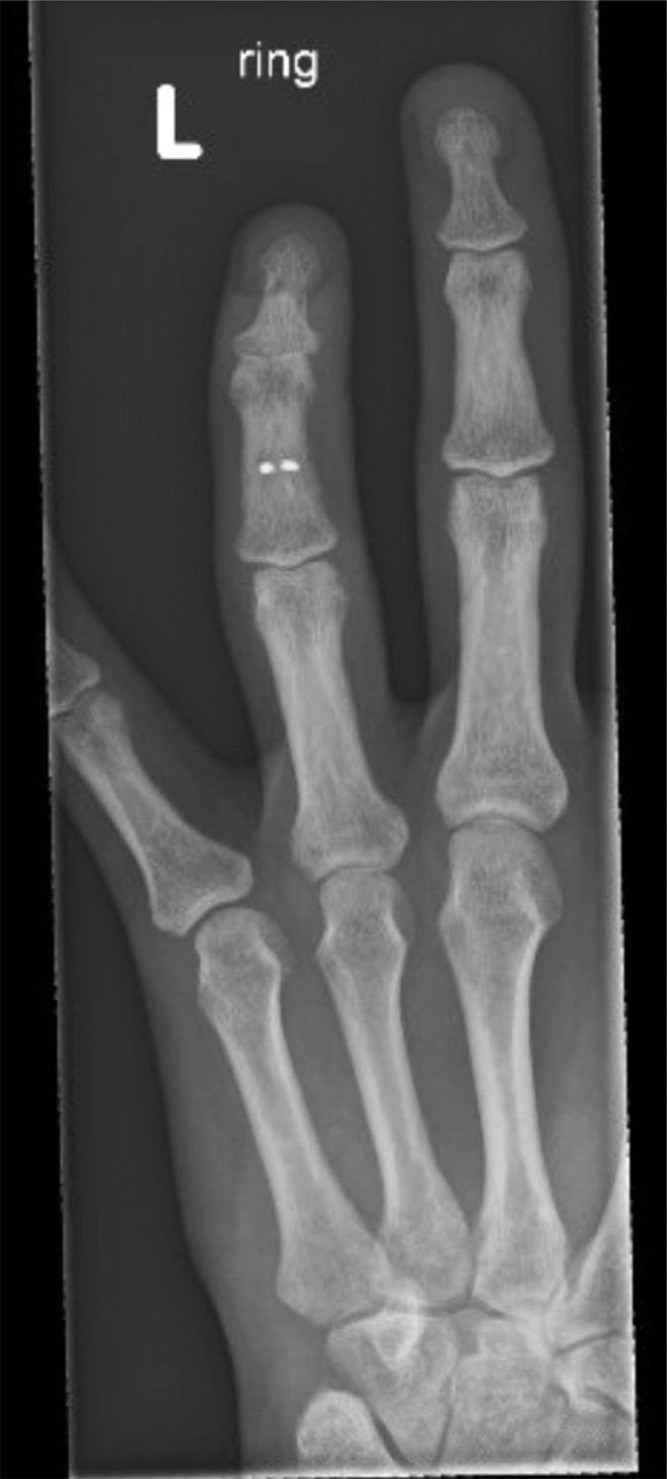
Frontal X-Ray of right hand (Case 2: Post-op).

**Figure 6.2:**
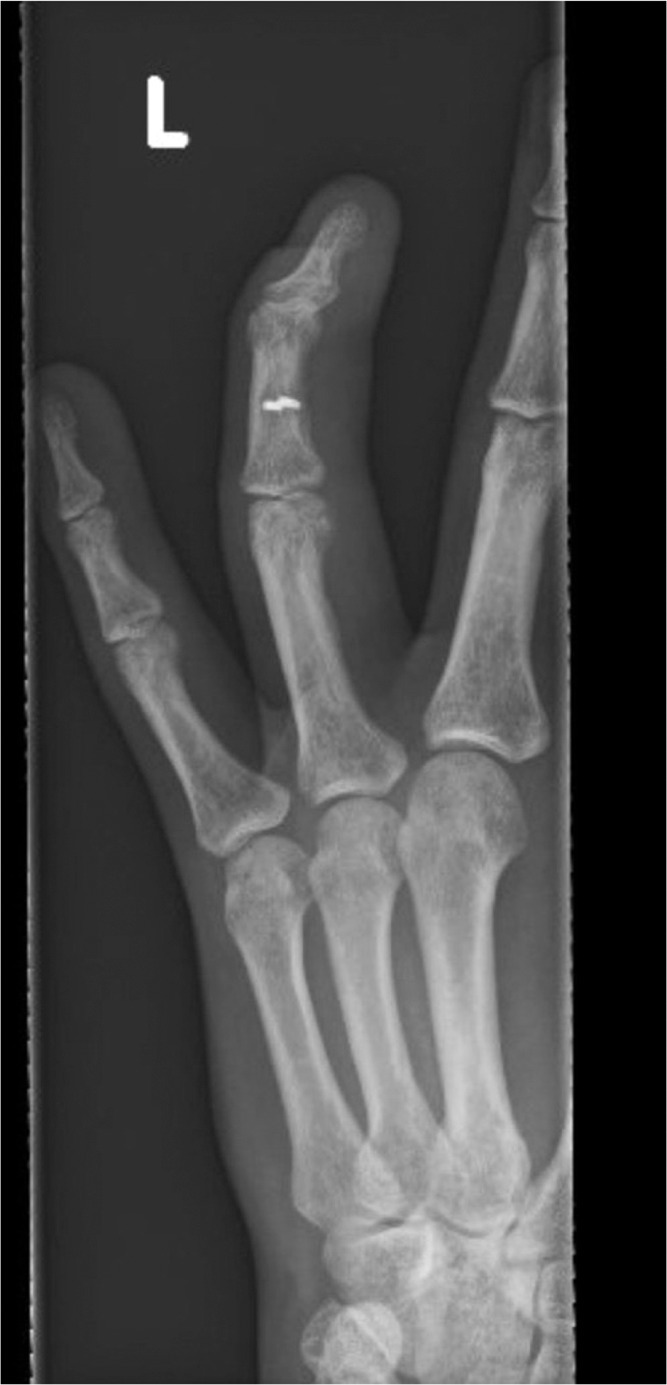
Oblique X-Ray of right hand (Case 2: Post-op).

**Figure 6.3:**
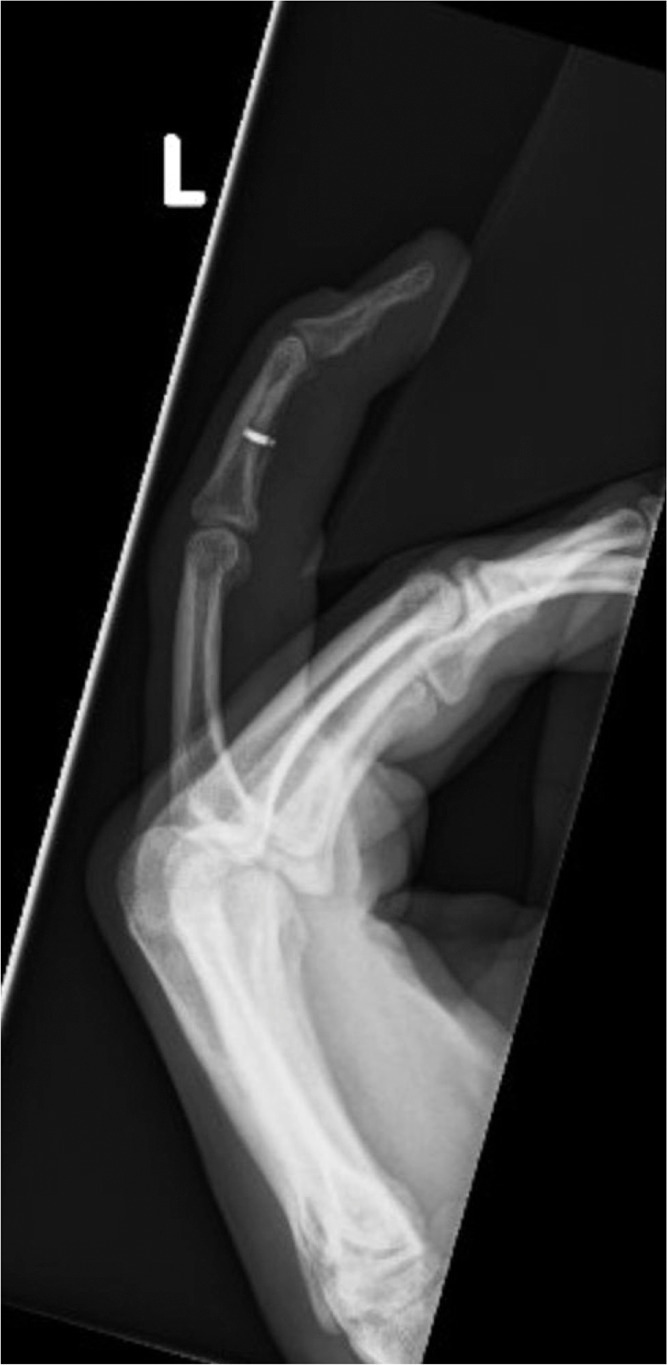
Lateral X-Ray of right hand (Case 2: Post-op).

During surgery, findings included a Leddy and Packer Type I FDP avulsion, avulsion of the FDS slip from its insertion, and disruption of the A5 and A4 pulleys, with the A2 pulley remaining intact. A standard Brunner zig-zag incision was made extending from the level of the A5 pulley proximally toward the A2 pulley to provide full exposure of the flexor sheath. After identification of the proximal tendon ends, the hematoma at the level of the A2 pulley was evacuated, followed by synovectomy and limited debridement of devitalized tissue. Partial release of scarred vincula and adhesions was performed as needed to mobilize the tendon stumps for repair. The FDS was repaired using two 3–0 mini Mitek anchor sutures. The FDP was repaired using a transosseous technique with 3–0 Prolene in a four-strand Adelaide configuration, secured over a button on the nail plate, allowing strong passive range of motion. The repair was reinforced with 4–0 PDS epitendinous sutures to the adjacent periosteum. The wound was closed with 4–0 nylon sutures (Figure 5.1, Figure 5.2, Figure 6.2–5.3) and post-operative X-Rays were taken to confirm fixation (Figures. 6.1–6.3). Postoperative dressing included Jelonet, gauze, crepe bandage, and a dorsal blocking splint. The patient was reviewed at 1 week, 2 weeks, 6 weeks, 3 months, and 6 months. At final follow-up, active range of motion values were: MCPJ 0–85°, PIPJ 0–100°, and DIPJ 0–65°, with a mild 5° extension lag at the DIPJ. The total active motion was 245–250°, equivalent to approximately 95 % of expected normal ROM. The patient experienced only minimal terminal stiffness at the DIPJ and successfully returned to unrestricted manual duties without functional impairment.

## Results

The PRISMA flow diagram (Supplementary Figure 1) began with 70 records identified, including 67 from databases and 3 from registers. After removing 7 duplicates, 63 records remained, of which 26 were excluded for measuring unintended outcomes and 1 was removed for not meeting the age limit, leaving 36 for screening. An additional 14 were excluded for focusing solely on single tendon ruptures. Of the 22 studies left for eligibility assessment, 1 was removed due to translation issues, and 5 because the full texts could not be obtained. This resulted in 16 studies (25 affected digits, 23 patients) being included in the systematic review (Table 1).^1^^,^^3^, ^4^, ^5^, ^6^, ^7^, ^8^, ^9^, ^10^, ^11^, ^12^, ^13^, ^14^, ^15^, ^16^, ^17^, ^18^

The age range was 16 to 79 years. The most commonly injured digit was the ring finger (44.0 %, 11/25), followed by the little (24.0 %, 6/25), middle (16.0 %, 4/25), and index fingers (8.0 %, 2/25). The most frequent mechanism was hyperextension or Jersey finger (60.0 %, 15/25), while blast, laceration, and crush injuries accounted for the remainder.

The most common site of FDP rupture was zone 1 (64.0 %, 14/25), followed by zone 2 (32.0 %, 8/25) and zone 3 (4.0 %, 1/25). FDS injuries occurred most frequently in zone 2 (48.0 %, 12/25), with zone 3 (20.0 %, 5/25) and zone 1 (16.0 %, 4/25) being less common. One case involved FDS rupture across zones 2 and 3.

All cases underwent surgical repair, with 64.0 % (16/25) receiving direct FDP reinsertion at its insertion. Pull-out sutures (36.0 %, 9/25) and anchor sutures (12.0 %, 3/25) were the most common fixation methods. FDS repair was performed in 44.0 % (11/25), while 48.0 % (12/25) underwent FDS resection. Two-stage tendon reconstruction with a tendon graft was performed in 8.0 % (2/25). The median time to surgery was 4 days (range: 0–60 days).

Across the 17 included studies (24 adult patients, 26 digits), postoperative functional recovery was variably reported, but several consistent patterns emerged. Total active motion was documented in eight cases, ranging from 230° to 260°, which represents good to excellent functional recovery in flexor tendon injuries. Joint-specific motion was also reported in multiple studies: MCPJ flexion typically ranged from 80 to 100°, PIPJ flexion from 85 to 105°, and DIPJ flexion from 20 to 70°, with occasional mild terminal extension lags (0–10°) at the PIPJ or DIPJ.

Complication rates were low. Reported issues included mild DIPJ stiffness (Backe & Posner; Oğün et al.) and small extension deficits (Johnson & Colville; Soro et al.; Naohito et al.), but no cases described tendon re-rupture or anchor pull-out. Most patients achieved functional use of the finger, commonly described as full ROM at the MCPJ and PIPJ, and adequate or improving DIPJ motion.

Follow-up ranged from 1.5 to 19 months. Most patients regained near-full range-of-motion (ROM), though some had residual stiffness, particularly at the DIP. Outcomes were best in cases treated within 1 week, whereas delays were associated with DIP and PIP stiffness.

## Discussion

Simultaneous closed rupture of the FDP and FDS tendons is exceedingly rare. The first case involved a delayed presentation following an altercation, where forced hyperextension of a flexed finger resulted in a closed avulsion of both tendons, with the retracted stumps found at the level of the MCPJ. The second case involved an acute sports-related injury in which the patient’s finger became entrapped in an opponent’s jumper, leading to an immediate dislocation and subsequent tendon avulsion. Despite the differences in timing, both cases underwent successful surgical repair, demonstrating the feasibility of primary repair even weeks after injury.

FDP avulsion injuries have been classically described by Leddy and Packer, with the classification including several subtypes based on the level of tendon retraction and associated injuries.² The first case was characterized by the simultaneous avulsion of the FDP and FDS tendons without bony injury, suggesting that an additional classification subtype is needed. We propose that this injury, similar in rarity to the type V injury, warrants inclusion as a separate entity in the Leddy and Packer classification as a “type VI” injury. Cuggy et al. have similarly suggested the need for a distinct category to account for combined flexor tendon avulsions, reinforcing the importance of recognizing this injury pattern.^15^ Given the involvement of both flexor tendons, this injury likely results from a unique biomechanical mechanism that differs from conventional FDP avulsions, necessitating specific operative considerations such as dual-tendon reinsertion techniques and strategies to restore appropriate tendon tension. Additionally, classification of this injury as a distinct entity may facilitate improved clinical documentation, enable comparative studies on surgical outcomes, and refine rehabilitation protocols to optimize functional recovery.

Using ultrasound as an adjunct to diagnosis in our case, while not definitive, allowed for a more precise preoperative surgical strategy. While ultrasound has shown positive results in diagnosing flexor tendon ruptures and has been extensively studied for single flexor tendon injuries, its effectiveness for multiple closed tendon injuries has yet to be fully established.^20^, ^21^, ^22^ Only two other authors have used ultrasound as an adjunct. While they also found it not definitive for diagnosis, they differed in reporting that it did not assist in defining their preoperative surgical strategy.^6^^,^^15^

The literature reveals a spectrum of surgical techniques tailored to tendon injury and zone of rupture. Soro et al. described an approach involving Z-plasty lengthening combined with a pull-out technique for the FDP and reinforcement with a palmaris longus tendon graft for the FDS, achieving favorable outcomes in a patient with zone 1 and zone 3 ruptures.^11^ Similarly, Cuggy et al. proposed modifications to the Leddy and Packer classification, incorporating cases of simultaneous FDP and FDS avulsions, highlighting the efficacy of early mobilization protocols post-repair.^15^ These studies underscore the importance of customizing repair techniques to the specific injury pattern to optimize functional recovery.

The choice to repair both tendons when FDP and FDS avulsions occur concurrently is debated among specialists. Some argue for FDS repair to achieve independent PIPJ flexion and enhance grip strength. Conversely, others warn that this approach might elevate the risk of tendon adhesion, which could limit the range of motion and require subsequent tenolysis. The only evidence for this is a study involving a small patient group with tendon injuries in zone 2C.^19^ In this case series, the FDS tendons were repaired because the injuries were situated in zone 3, and the flexor sheath and pulley system remained intact proximal to the insertion.

In terms of surgical techniques, approximately half of the authors opted to resect the FDS tendon, particularly in cases with avulsion fractures or where the FDS was ruptured at its insertion site. The remaining authors employed various repair techniques, including pull-out sutures, periosteal flaps, tendon grafts, and suture anchors. Our approach utilized micro-Mitek suture anchors for the FDP and FDS tendons, which another case report also adopted.^15^ Micro-Mitek suture anchors for flexor tendon repairs offer increased strength and potentially faster return to function compared to traditional pull-out suture methods.^23^^,^^24^ This technique facilitates early active mobilization, reduces the risk of adhesion formation, and promotes better tendon gliding. The enhanced strength of the suture anchors allows the tendons to withstand higher tensile loads compared to traditional suture techniques, leading to a reduced risk of repair failure and faster functional recovery.^23^^,^^24^ Compared to tendon grafting, suture anchors eliminate the need for graft harvesting, thereby reducing surgical complexity and associated morbidity.^25^ Overall, micro-Mitek suture anchors offer a robust, efficient, and patient-friendly alternative to traditional tendon repair methods.

Outcomes across the cases varied, but many reported good to full recovery of range of motion at the MCP, PIP, and DIP joints. The use of suture anchors and early mobilization protocols were associated with favorable outcomes, including full ROM and minimal stiffness (Table 1). However, some cases still experienced complications such as stiffness and reduced flexion, particularly at the DIP joint.^26^^,^^27^ These findings underscore the importance of selecting the appropriate surgical technique based on the specific injury characteristics and ensuring rigorous postoperative rehabilitation to optimize functional recovery.

Additionally, the literature suggests that while resection of the FDS tendon can reduce the risk of adhesions and simplify the surgical procedure, it may compromise the strength and independent flexion of the PIP joint.^28^^,^^29^ Conversely, repairing both tendons can preserve more natural finger mechanics but increases the complexity of the surgery and the risk of postoperative complications. Our case, utilizing suture anchors, demonstrated excellent intraoperative tendon glide and early mobilization, supporting the argument for their use in suitable cases. Moving forward, the development of standardized protocols for the surgical management and rehabilitation of combined FDP and FDS ruptures could further enhance patient outcomes and reduce variability in treatment success.

To enhance the reliability of this case series, we systematically reviewed all published reports of simultaneous FDP and FDS tendon avulsions. The primary outcomes we assessed included the mechanism and timing of injury, the zone of tendon rupture, the surgical technique employed, and postoperative functional recovery—including total active motion, ROM at individual joints, and complication rates (e.g., adhesions, re-rupture, stiffness). It is important to acknowledge potential biases within the literature. All 17 reviewed studies are isolated case reports or small case series, often involving young, active males and lacking standardized outcome reporting. Selection and reporting biases may therefore limit the generalizability of observed outcomes. Furthermore, variation in surgical expertise and rehabilitation protocols introduces heterogeneity that complicates direct comparisons. While we initially commented on the technical challenges of dual-tendon repair, a broader perspective on study quality is equally crucial. The overall low level of evidence, absence of comparative cohorts, and inconsistent reporting highlight a clear need for prospective multicenter studies with standardized outcome measures. Such studies could establish best practices for managing these rare but functionally significant injuries.

## Conclusion

This case series contributes to the limited body of knowledge on simultaneously closed avulsions of both the FDP and FDS tendons. It demonstrates that delayed primary repair can yield successful outcomes, advocating for a flexible approach to surgical timing and technique. The importance of individualized surgical planning and comprehensive postoperative rehabilitation is paramount in achieving optimal functional recovery. Further research and accumulation of similar case reports are essential to refine the management strategies for such rare and complex injuries in hand surgery.

## Patient consent

Patients/guardians have given informed consent to the publication of images and/or data.

## Financial disclosure

No authors have received any funding or financial support.

## Declaration of competing interest

The authors declare no conflict of interests.

## Appendix Supplementary materials

Image, image 1 dummy alt text

Supplementary Figure 1. 2020 PRISMA Flowchart Diagram. Image, application 2
