# Supplementary figures and images for: Double jersey finger: A systematic review and case series
Source: JPRAS Open. 2026 Mar 16;49:402–18. doi: 10.1016/j.jpra.2026.03.005 (PMC13068791; doi:10.1016/j.jpra.2026.03.005)

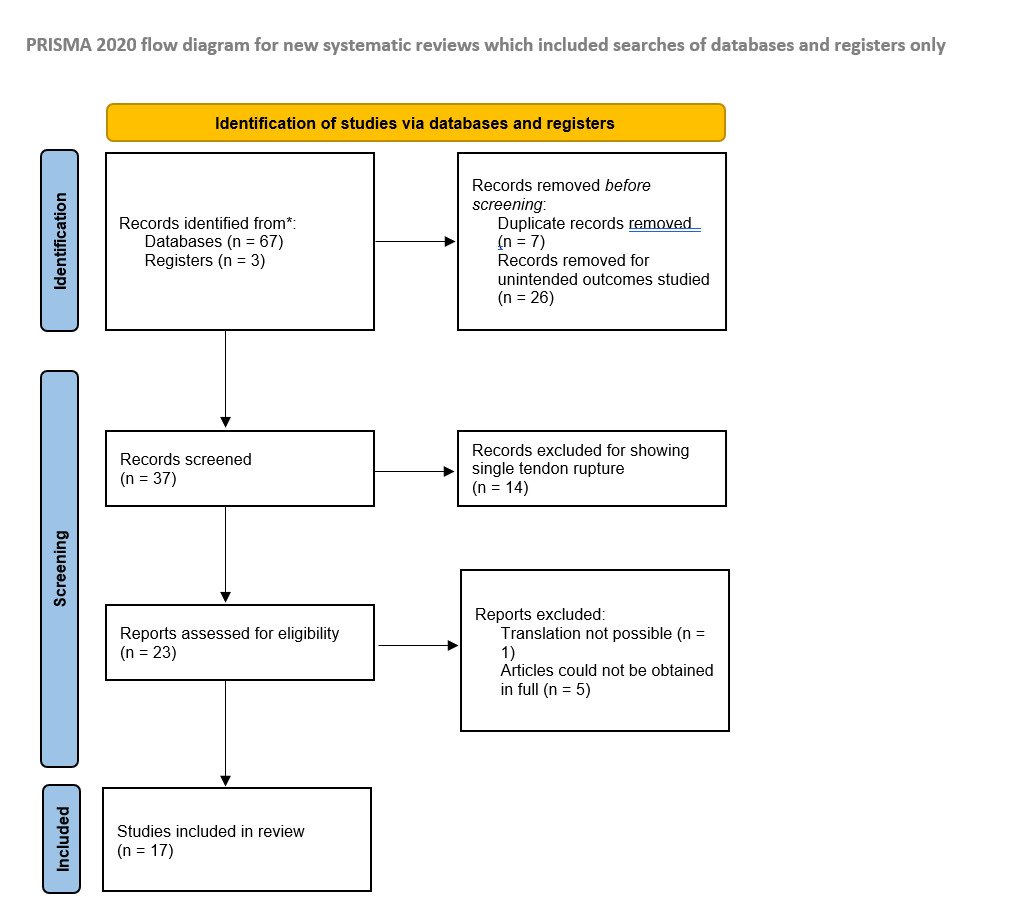

Supplement: Supplementary file 1 [file mmc1.jpg]
