# Supplementary material for: Double jersey finger: A systematic review and case series
Source: JPRAS Open. 2026 Mar 16;49:402–18. doi: 10.1016/j.jpra.2026.03.005 (PMC13068791; doi:10.1016/j.jpra.2026.03.005)
Supplement: Supplementary file 2 — Supplementary Figure 1. 2020 PRISMA Flowchart Diagram. [file mmc2.docx]

**Identification of studies via databases and registers**

Records removed *before screening*:

Duplicate records removed (n = 7)

Records removed for unintended outcomes studied (n = 26)

Records identified from*:

Databases (n = 67)

Registers (n = 3)

**Identification**

Records excluded for showing single tendon rupture

(n = 14)

Records screened

(n = 37)

**Screening**

Reports excluded:

Translation not possible (n = 1)

Articles could not be obtained in full (n = 5)

Reports assessed for eligibility

(n = 23)

Studies included in review

(n = 17)

**Included**
